# The role of toothbrush in the transmission of corona- and influenza viruses — results of an in vitro study

**DOI:** 10.1007/s00784-022-04530-w

**Published:** 2022-05-10

**Authors:** Gerhard Schmalz, Laura Feindt, Franziska Tanneberger, Rainer Haak, Ahmed Abd El Wahed, Uwe Truyen, Dirk Ziebolz

**Affiliations:** 1 Department of Cariology, Endodontology and Periodontology, University of Leipzig, Liebigstr. 12, 04103 Leipzig, Germany; 2 Institute for Animal Hygiene and Veterinary Public Health, Faculty of Veterinary Medicine, University of Leipzig, 04103 Leipzig, Germany

**Keywords:** Toothbrush, Oral hygiene, Coronavirus, Influenza, Transmission

## Abstract

**Objectives:**

The aim of this in vitro study was to investigate viruses’ stabilities on manual toothbrushes using feline coronavirus (FeCoV) as representative of coronaviruses and an Avian influenza A virus H1N1 for influenza viruses.

**Material and methods:**

Two viruses, FeCoV (Strain Munich; titer 107.5 TCID50/ml) and H1N1 (RE 230/90; titer 106.5 TCID50/ml), were used in this study. Manual toothbrushes were disassembled into bristles, bristle fixation, and back of the toothbrush head, contaminated with the viruses and air-dried for 24 h. In a second experiment, whole toothbrush heads were contaminated, rinsed with water (5 ml for 15 s) and then air-dried.

**Results:**

For FeCoV, immediately after contamination, the following average titers were recovered: fixation: 10^6.41^, back of head: 10^6.81^ and bristles: 10^6.63^ TCID_50_/ml. Following air-drying of 12 (fixation) and 24 h, titers of ≤ 10^2.5^, 10^3.75^, and 10^2.72^ TCID_50_/ml were found in the respective groups, with a detection limit of 10^2.5^ TCID_50_/ml. For H1N1, immediately after contamination, the following average titers could be recovered: fixation: 10^5.53^, back of head: 10^5.97^ and bristles: 10^5.75^ TCID_50_/ml. Following air-drying of 8 (fixation) and 24 h, titers were ≤ 10^2.5^, 10^3.63^, and 10^3.53^ TCID_50_/ml in the respective group, again with 10^2.5^ TCID_50_/ml being the detection limit. In case of water rinse, no infectious virus could be recovered after 12 h.

**Conclusion:**

Viral load of both viruses is reduced by air-drying, especially following water rinsing.

Clinical relevance

The toothbrush itself plays an insignificant role in the self-transmission of coronavirus and influenza virus.

## Introduction

During the past 2 years, a viral pandemic with a novel human coronavirus (severe acute respiratory syndrome coronavirus 2, SARS-CoV-2) lead to a global public health crisis. This circumstance caused a high impact on daily life, including dentistry and oral hygiene issues [1]. The virus SARS-CoV-2 is mainly located in the nasopharyngeal tract as a main source for transmission, while the oral cavity and saliva also contains a certain amount of viral load, which is, however, of little value for airborne transmission of the virus [2]. Accordingly, oral hygiene issues were repeatedly and comprehensively discussed in context of the current pandemic situation. On the one hand, usage of mouthwashes to reduce the viral load, and thus, the risk of transmission is an issue of high interest [3]. On the other hand, oral hygiene aids were reported as potential habitat for SARS-CoV-2, increasing the risk of transmission. Thereby, oral hygiene aids were reported as potential way to spread the SARS-CoV-2 infection to cohabitating individuals [4]. However, until now, there is no evidence on the transmission risk of viruses via oral hygiene aids.

Generally, the toothbrush has been examined as a potential source of microorganisms in different studies, including both bacterial and fungal species [5–8]. While toothbrushes are commonly used for daily oral hygiene, their potential risk as a source of infection opens a new view on a potentially neglected health risk [9, 10]. Toothbrushes of both, healthy and diseased individuals, become contaminated with oral bacteria, especially originating from dental plaque [9]. Furthermore, other bacteria or fungi might also colonize the toothbrush, serving as a habitat for (self-) infection [11]. Therefore, a variety of disinfection approaches to remove microorganisms from toothbrushes have been developed and evaluated, including microwave cooking, vinegar, alcohol, oral antiseptics, and UV light [12–14]. Most of those approaches focused on the removal of bacteria from toothbrushes, showing mainly effective disinfection results [12–14]. However, against the background of the current pandemic situation, the potential necessity of disinfection of toothbrushes to reduce viral load would be of interest, too. As first step to answer this question, it would be essential to investigate, whether viruses would be detectable on toothbrushes in an amount, which could potentially lead to a (self-) transmission. In this context, the effect of water rinsing and air-drying, as commonly performed after toothbrushing, would be needed to be considered.

Accordingly, this in vitro study had two aims: (I) it was examined whether two different viruses of importance and known to be transmitted via respiratory aerosols, i.e., coronavirus and influenza virus, would be detectable in a clinically relevant load on different parts of the manual toothbrush, depending on the time of air-drying. In this study, the feline coronavirus and the avian influenza A virus H1N1 were used. (II) It was also tested, if rinsing with water could reduce the viral load on the manual toothbrush. For this, a common high-quality standard protocol for testing viral load was applied. To differentiate the findings, different areas of the toothbrush were examined. It was hypothesized that viral load would be detectable at all parts of the toothbrush, while both air-drying and water rinsing lead to elimination of the titer of both viruses.

## Materials and methods

### Viruses and cell cultures

In this study, FeCoV (strain Munich) was applied as a representative virus for SARS-CoV-2. Furthermore, an avian Influenza A Virus H1N1 (RE 230/90) virus was used as an analogue for a human influenza virus. FeCoV was propagated in Crandell Rees Feline Kidney (CRFK) cells to obtain a titer of 10^7.5^ TCID_50_/ml. AIV H1N1 was propagated in chicken embryo fibroblast to a titer of 10^6.5^ TCID_50_/ml. The cultivation of the viruses was conducted at 37 °C and 5% CO_2_. The experiments were performed separately with each of the two viruses to assess the respective characteristics of the viruses.

### Toothbrushes

Manual toothbrushes (*Dr.*BEST Original, CLASSIC; GSK Consumer Healthcare, D-80258 München, CH-6343 Rich) were bought from public shops and used as test material. They have standardized flat bristles and were selected in the hardness grade medium. For examination of the contamination, (I) toothbrushes were assembled inside the laminar flow cabinet class II under sterile conditions to the three parts: bristle fixation, the back of the toothbrush, and bristles, which have been investigated separately. (II) For the second experiment, the entire toothbrush head was used.

### Test procedure

#### Viral contamination of three different areas of the toothbrush with subsequent various incubation periods and titer determination

##### Contamination procedure

**Fig. 1:**
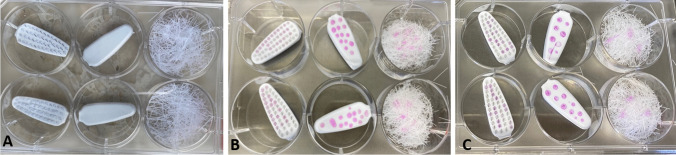
Preparation and incubation of the toothbrush parts. **A** Tissue culture plate (6-well) with prepared areas of the toothbrush; **B** 50 μl virus on each area of the toothbrush right after contamination; **C** 50 μl virus on each area of the toothbrush after 24 h of air drying

For the first experiment, the manual toothbrushes were disassembled to constitute the three parts: bristle fixation, back of toothbrush, and bristles themselves to determine the viral load on these areas at various time points. The various parts were contaminated with 50 μl of one of the two viruses and incubated for different periods of time (Fig. 1).

**Fig. 2:**
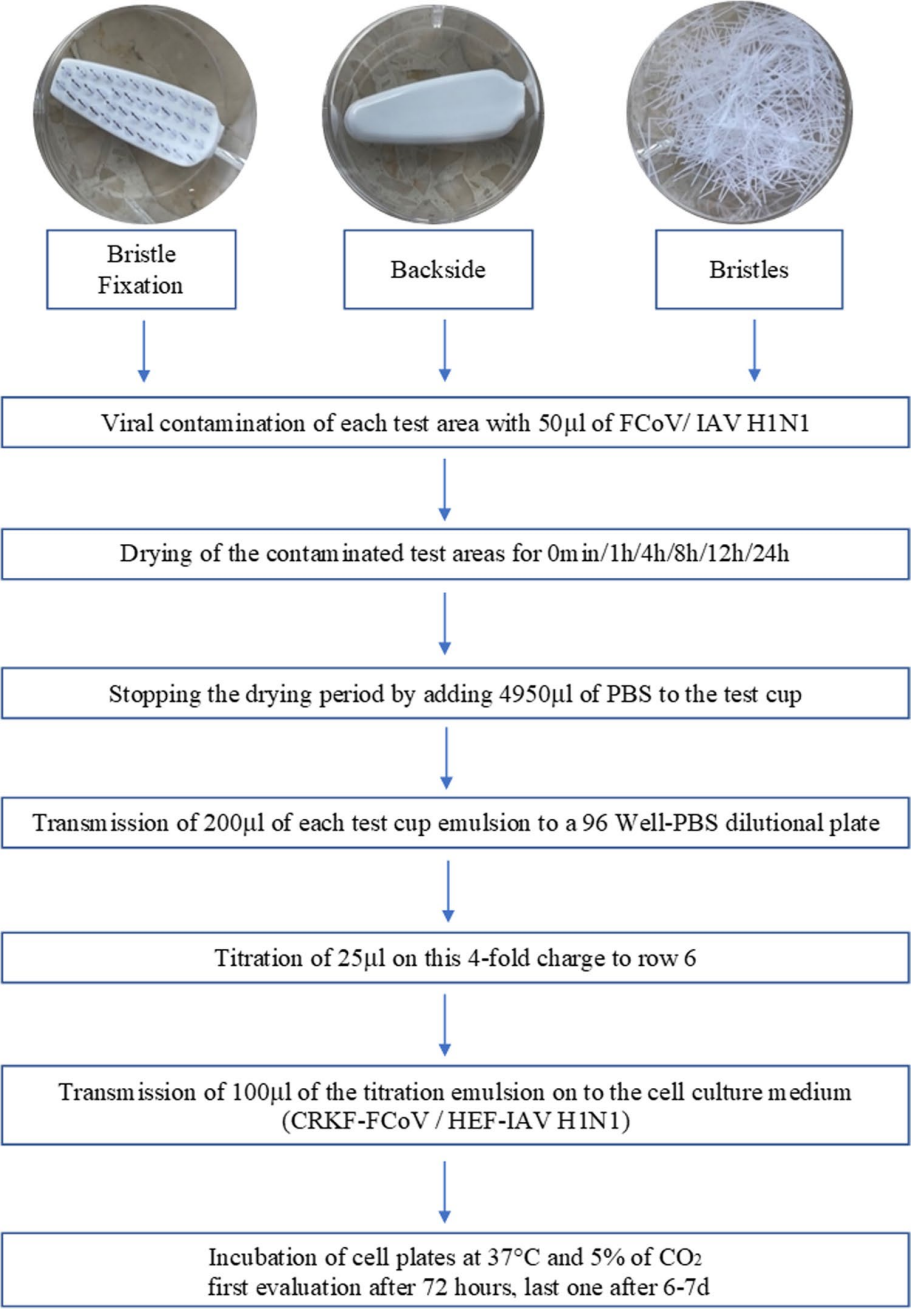
Experimental flow of the testing of different parts of the toothbrush

The viral load was ascertained immediately after contamination and after an incubation period of 1, 4, 8, 12, and 24 h. The examination areas contaminated with virus dried in a laminar flow cabinet at a room temperature of 23.5 °C on a 6-well plate (TC-plate 6-well, standard, F). For each time period, the proceeding was assessed with 8-fold repeats. The experimental flow is displayed in Fig. 2.

##### Quantification of viral tissue culture infective dose 50 (TCID50)

To recover the remaining virus on the toothbrush areas, 4950 μl of phosphate buffered saline (PBS) was added to each cup of the 6-well plate after each drying phase. The areas were washed 10 times with the PBS to ensure that the virus was completely suspended. Subsequently, 200 μl of each area were transferred to a 96-well-PBS dilution plate, and titrated in log_10_ steps. After 2 days, the cell plates were examined under an optical microscope to investigate the cells on a cytopathic effect as a verification for the existence of remaining virus. The cell observation was proceeded for about 7 days to determine the 50% tissue culture infectious doses (TCID50) according to the Spearman–Kaerber method.

#### Viral contamination of toothbrush with subsequent water rinsing and air-drying for 12 h

##### Contamination procedure

**Fig. 3:**
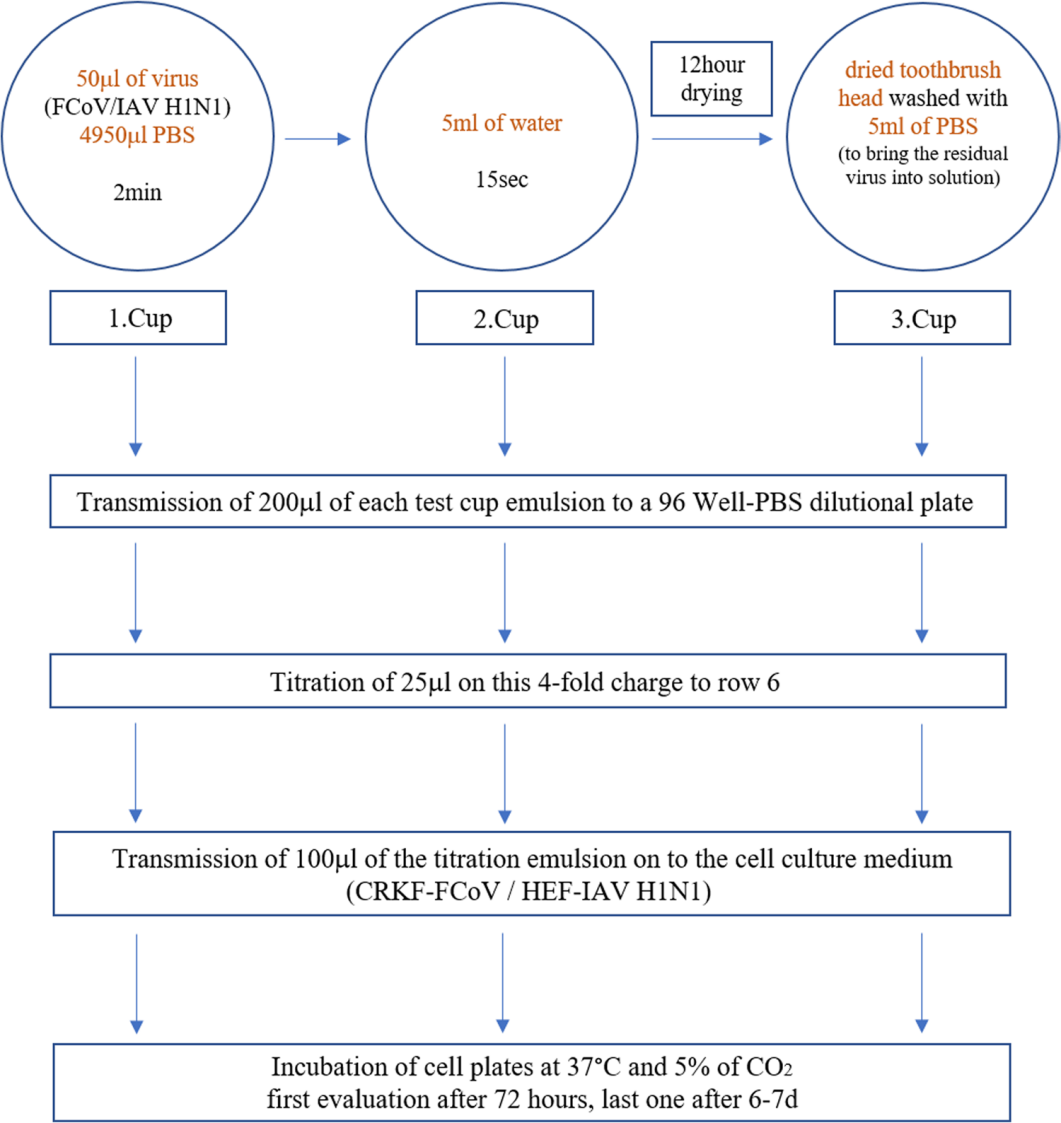
Experimental flow of the contamination of toothbrush with subsequent water rinsing and air drying

The experimental flow is shown in Fig. 3. To adapt the procedure of toothbrushing, the head of the toothbrush was approximately dipped in a cup of a 6-well plate with virus solution of 50 μl of either FeCoV or AIV H1N1 and 4950 μl PBS for 2 min. Afterwards the toothbrush was rinsed in the next cup filled with 5 ml of water for 15 s. The drying of the toothbrush head ensued in the third cup of the 6-well plate in a laminar flow cabinet at a room temperature of 23.5 °C for 12 h.

##### Quantification of viral tissue culture infective dose 50 (TCID50)

After the drying phase, 5 ml of PBS was added into the cup of the 6-well plate to rinse the toothbrush head and bring the remaining virus on the toothbrush in solution.

200 μl of each cup (virus solution, rinsing water, remaining virus on dried toothbrush head added with PBS) were transfused to a 96-well-PBS dilutional plate. The subsequent procedure was conducted as depicted above. 100 μl of each serial dilution was transfused to the appropriate cell culture system in each case.

For the FeCoV, Crandell Rees Feline Kidney (CRFK) cells were used; for IAV H1N1, chicken embryo fibroblasts (HEF) were utilized for cell culture. Thereafter, the plates were incubated at 37 °C and 5% of CO_2_. As described in the procedure above, the plates were examined under the microscope for 7 days to investigate the cells on a cytopathic effect as a positive verification for virus. By means of the results, the titer could be determined according to the Spearman–Kaerber method.

### Statistical analysis

All experiments were performed with 8-fold repeats. For statistical analysis, the software GraphPad-PRISM was used.

## Results

### Viral contamination of three different areas of the toothbrush with subsequent various incubation periods and titer determination

#### Contamination with FeCoV

**Fig. 4:**
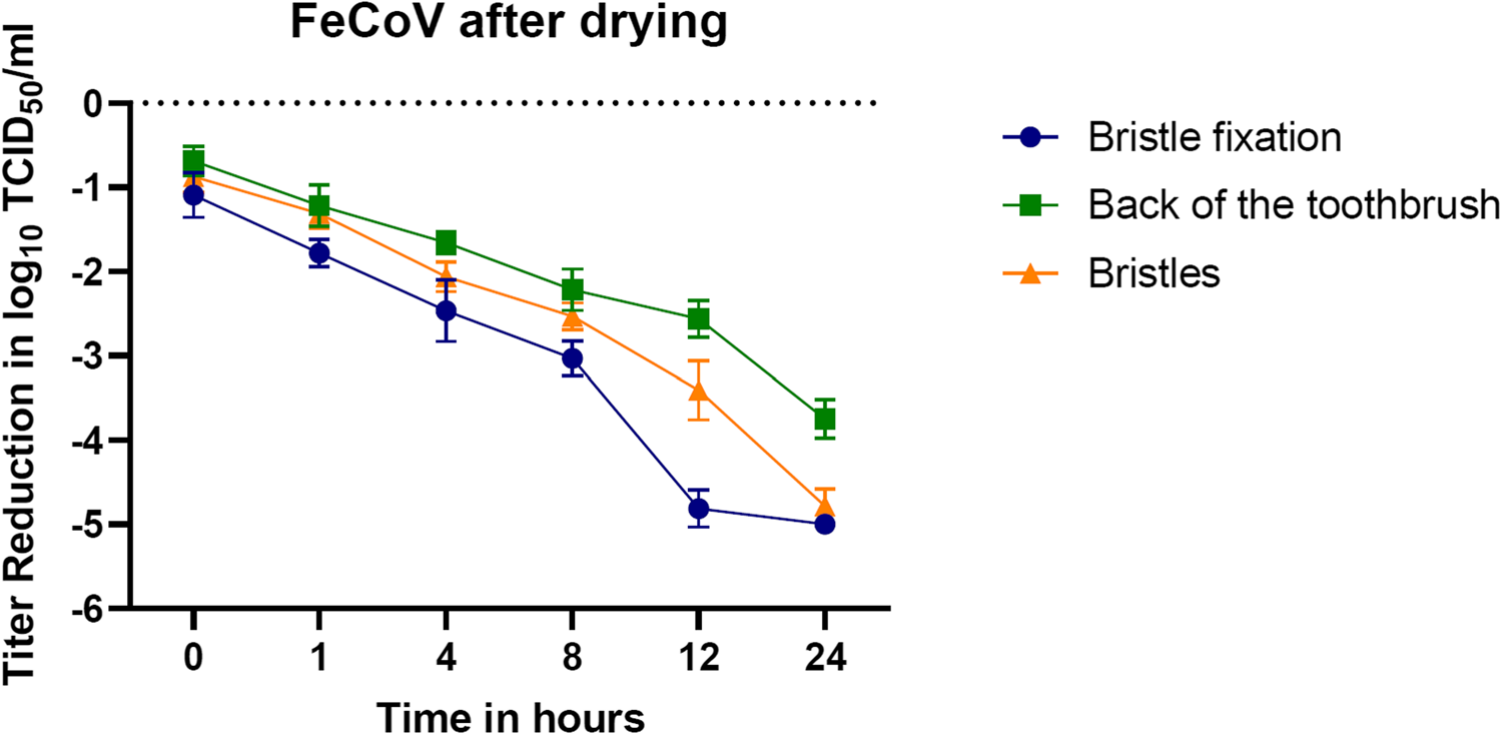
Reduction of the FeCoV titer after contamination during 24 h of drying. In case of identical results, error bars were not shown. The reduction rate was measured as the value of control viral titer minus the normalized value of the tested part of the toothbrush

The results of the first experiment displayed that the verified remaining viral burden on the toothbrush diminishes with increasing drying time and is dependent on the respective toothbrush part (Fig. 4). The FeCoV batch was determined with an output titer of 10^7.5^ TCID_50_/ml. Immediately after contamination, a titer loss of approximately 1 log_10_ level was detected in all three contaminated parts of the toothbrush. This titer-reducing tendency continued throughout the drying period. After only 12 h, no infectious residual virus could be detected on the bristle fixation. Little residual infectious virus was still detected on the back of the brush as well as on the bristles after 24 h of drying with titers of 10^3.75^ TCID_50_/ml and 10^2.72^ TCID_50_/ml, respectively. In some repetitions, the smallest detectable titer of ≤ 10^2.5^ TCID_50_/ml was determined for the bristles after 24 h of drying.

#### Contamination with IAV H1N1

**Fig. 5:**
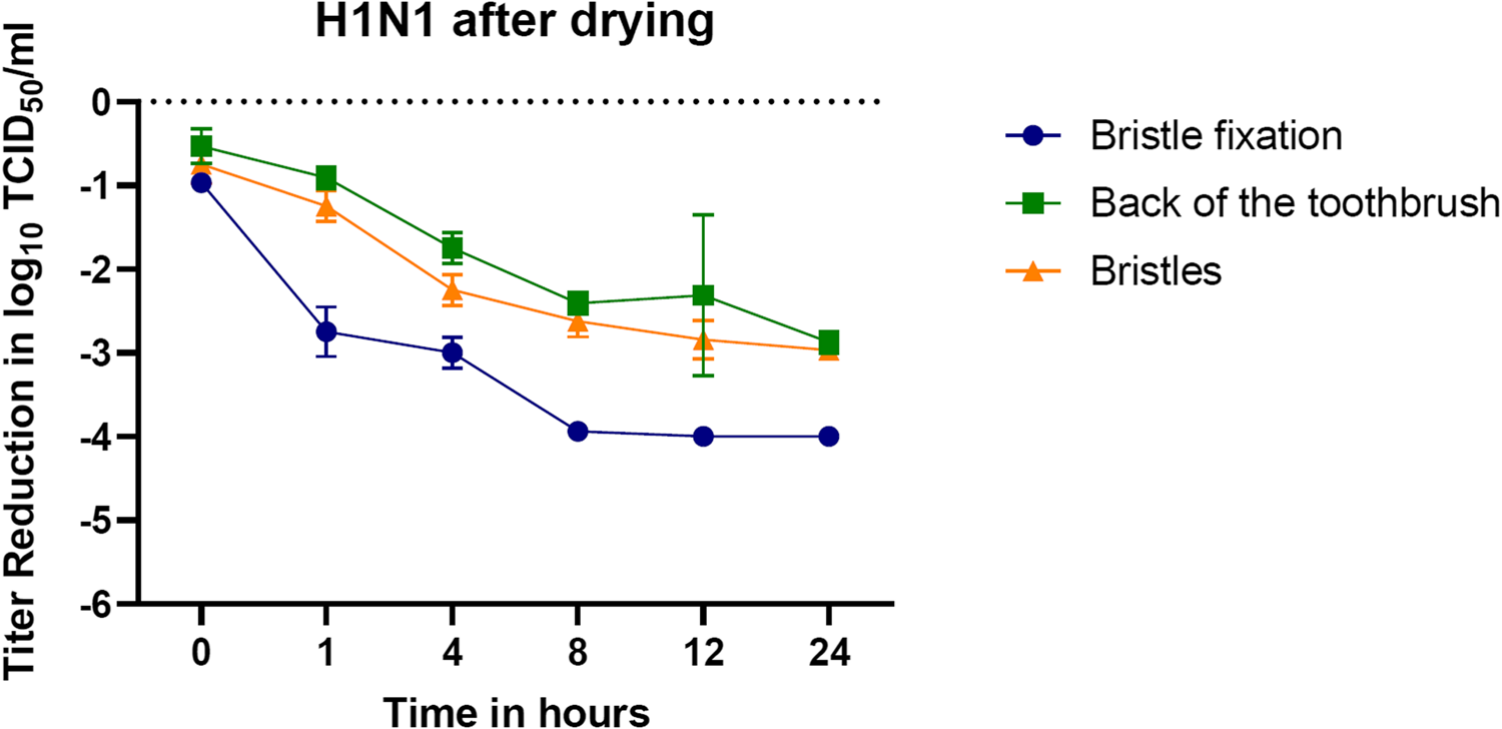
Reduction of the H1N1 titer after contamination during 24 h of drying. In case of identical results, error bars were not shown. The reduction rate was measured as the value of control viral titer minus the normalized value of the tested part of the toothbrush

It was evident in the experiment that the determined viral load decreases with an increasing drying phase. Moreover, the virus does not retain as long on the bristle fixation compared to the back of the toothbrush and the bristles (Fig. 5). The IAV H1N1 virus batch was determined with an output titer of 10^6.5^ TCID_50_/ml. Right after the contamination of the toothbrush areas, the titers of the remaining virus on all tested toothbrush areas declined by approximately 0.5–1 log_10_ levels. After only 8 h of drying, residual virus titers on the bristle fixation were reduced to just above the detection limit of ≤ 10^2.5^ TCID_50_/ml. In some repetitions, even a value below the detection limit was determined. The experimentally verified viral load on the back of the toothbrush and the bristles also diminished with increasing drying phase, but lasted longer on these areas. After 24 h of drying, the remaining titers on the back of the toothbrush and the bristles were recorded with approximately a 3 log_10_ loss.

### Viral contamination of toothbrush with subsequent water rinsing and air drying for 12 h

#### Contamination with FeCoV

**Fig. 6:**
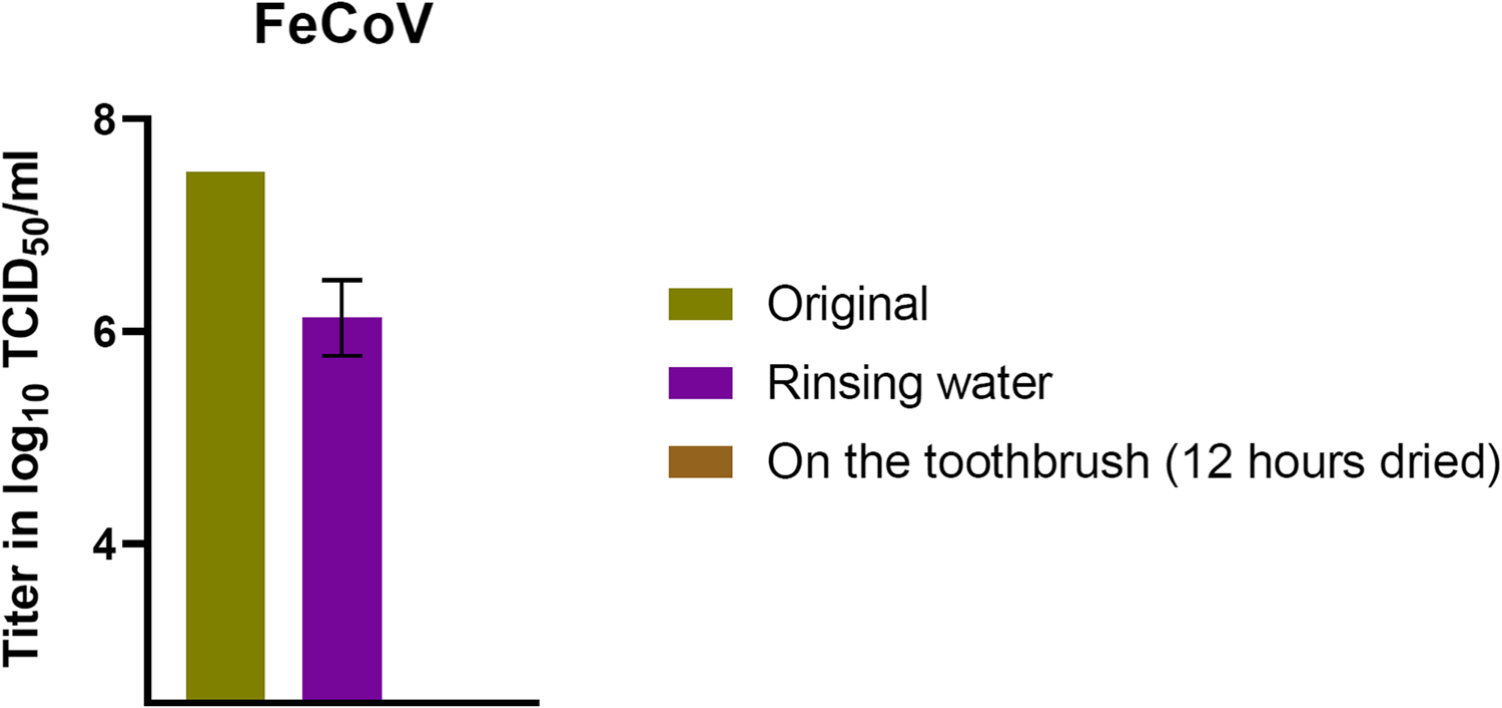
Viral load of FeCoV after water rinse and air-drying for 12 h (limit of detection ≤ 10^2.5^ TCID_50_/ml). The control viral load was identical; therefore, no error bars were shown

The rinsing of the toothbrush, followed by a drying period of 12 h, showed a reduction of the viral load (Fig. 6). The average titer of the viral solution, in which the toothbrush head was dipped in, was measured with a value of 10^7.16^ TCID_50_/ml. The remaining virus in the rinsing water led to a mean titer of 10^6.13^ TCID_50_/ml. After a drying time of 12 h, a titer drop below the detection limit was determined on the toothbrush head.

#### Contamination with IAV H1N1

**Fig. 7:**
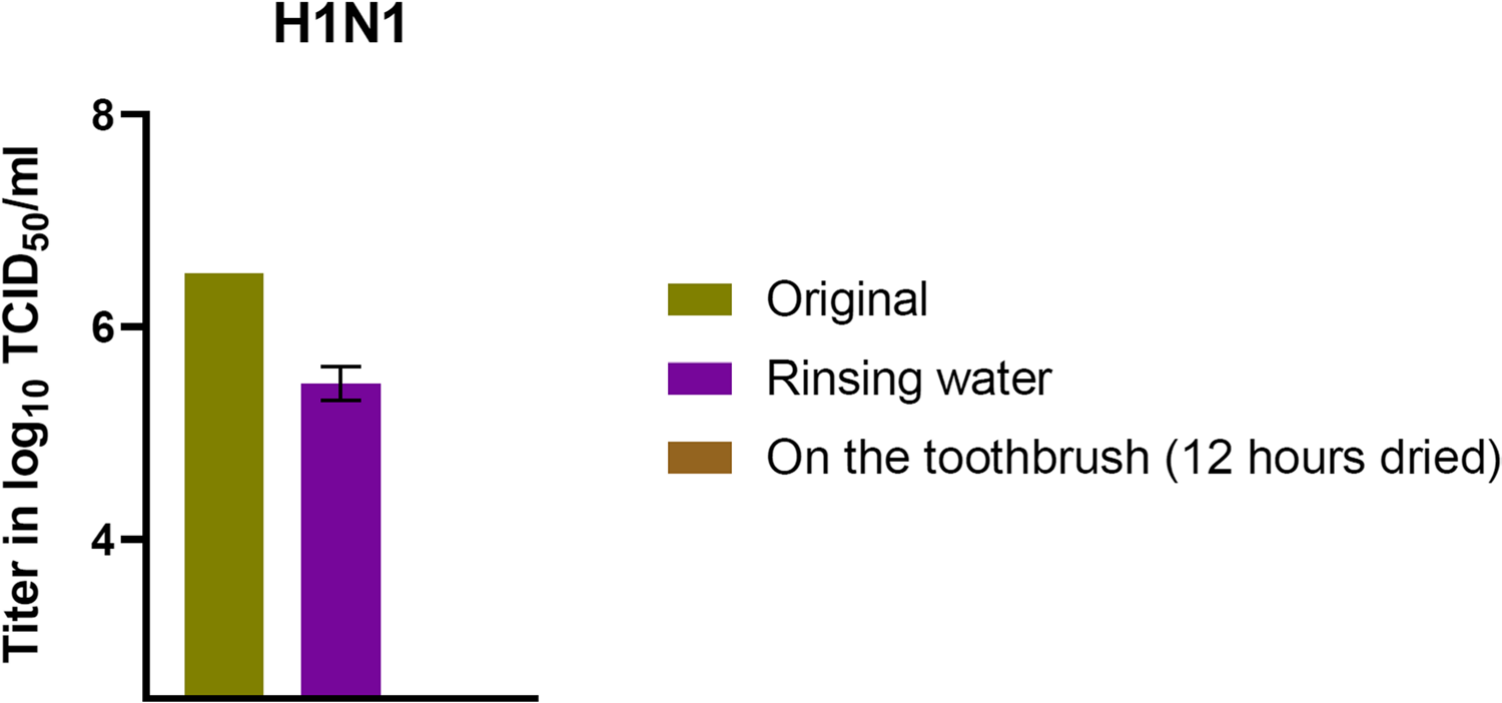
Viral load of H1N1 after water rinse and air-drying for 12 h (limit of detection ≤ 10^2.5^ TCID_50_/ml**).** The control viral load was identical; therefore, no error bars were shown

As in the case of FeCoV contamination, it was also evident in the case of contamination with IAV H1N1 that rinsing the toothbrush and subsequent air-drying for 12 h resulted in a reduction of the viral load (Fig. 7). The viral solution, in which the toothbrush head was dipped in, was measured with an average titer of 10^6.23^ TCID_50_/ml. A mean value of 10^6.23^ TCID_50_/ml was determined in the rinsing water. After a drying time of 12 h, a titer drop below the detection limit was determined on the toothbrush head.

## Discussion

The current worldwide pandemic situation shows the danger of the speed of spread of viral pathogens, which should not be underestimated. In the current event of a respiratory virus such as SARS-CoV-2, potential spread cycles must also be recognized on an everyday scale. In this context, dental hygiene plays a role, in addition to the already generally established measures such as personal hand hygiene. Aside of this way of infection, the transmission via contaminated surfaces has been thoroughly discussed [15]. In order to assess the stability of important respiratory viruses on products of dental hygiene like toothbrushes and the associated (re)infection risk, contamination experiments with a coronavirus (FeCoV) and an influenza virus (AIV H1N1) were performed in this study.

For this purpose, controlled contamination experiments of different toothbrush areas (bristles, back or fixation) were performed to analyze virus tenacity. It was found that the titers of both viruses were rapidly and steadily reduced over the 24 h of the experiment. This reducing effect was particularly rapid on the contaminated toothbrush fixation. Furthermore, already within 12 h, an effective titer reduction of 2.5–5 log_10_ (FeCoV) and 2–4 log_10_ (H1N1) could be detected on all tested toothbrush parts. The residual titer was just above the detection limit at this point and changed little over the remainder of the experiment. Thus, both viruses show a low stability, which is further reduced by rinsing of the contaminated toothbrush parts, where no active virus could be recovered.

Meanwhile, different studies report a certain stability of the infectivity of coronaviruses, especially SARS-CoV-2 on surfaces [15]. Interestingly, a previous study showed coronaviruses to remain infective on plastic surfaces (which is also the material of most toothbrushes) for hours, showing viable virus for up to 72 h after application [16]. However, this previous examination showed very low titers after such a long observation period, while the titers after 12 and 24 h were similarly low as in the current study [16]. Therefore, although a certain stability of the virus was detectable, the infectivity of the contaminated surface is very low, making a transmission unlikely. In case of the current study, the air-drying at room temperature seems to lead to a remarkable and fast reduction of the titer (i.e., below the limit of detection). Another study showed that the stability of coronavirus is remarkable reduced at 20 °C [17]. Thus, the room temperature and laminar flow appear to lead to fast evaporation of the droplets and thus decreased viral load. Causal for this, the alteration of the envelope of the coronavirus because of continuous air-drying would be a plausible explanation for the fast loss in its infectivity. With regard to the clinical reality, a toothbrush is regularly rinsed with water after use. This was an experiment in the current study, resulting in a complete loss of virus load after rinsing and subsequently 12 h air-drying. As a result, the toothbrush is no habitat with a high risk of self-infection. It seems more plausible that patients, using the same toothbrush transmit the virus to each other, because of a generally reduced health behavior, for which using the same toothbrush could be an indicator. In this context, patients regularly use toothpaste for toothbrushing; toothpaste has an antimicrobial effect; although this was mainly shown for bacteria, the influence of the contamination of the toothbrush remains unclear [18, 19].

FeCoV was selected as an alternative test virus for SARS-CoV-2 in this study. Both viruses belong to the family of *Coronaviridae*; those viruses have an enveloped spherical structure with a diameter between 60 and 160 nm, while Influenza virus belongs to the family *Orthomyxoviridae*, having an enveloped pleomorphic structure with a diameter ranging from 100 to 120 nm [20]. This so-called surrogate virus method has long been used for efficacy testing of chemical disinfectants according to the guidelines of the German Veterinary Society (DVG). For this purpose, FeCoV and other viruses are commonly used as surrogates for related viruses and the results are directly transferred to the original viruses.

In addition to a coronavirus, the current experiment was also performed with another respiratory virus with high clinical relevance: Avian Influenza A virus H1N1. AIV H1N1, which caused a pandemic in 2009 [21], is a type A influenza virus, an enveloped RNA virus, having a pleomorphic appearance with an average diameter of 120 nm [22]. Influenza viruses are of high clinical interest, as they have caused hundreds of thousands of deaths worldwide each year [23]. Moreover, in the current pandemic, influenza and SARS-CoV-2 are co-existing viruses, needing a joint preventive approach [24]. Against this background, the same research question was applied for H1N1 in the current investigation, showing comparable results as for FeCoV. In turn, a study by Oxford et al. found that H1N1 is still infectious after 24 h on a plastic surface [25]. During this study, H1N1 showed slightly lower titer reduction in the air-drying experiment than FeCoV; however, the titer after 12 and 24 h was low. Furthermore, the results after water rinse, again, corresponding to the clinical situation, were equal. Therefore, the toothbrush was also identified to be no important source of H1N1 and thus probably influenza virus (self-) transmission.

In general, a higher viral load of both viruses on bristles and bristle fixation would have been expected, based on the higher surface size. Nevertheless, the back of toothbrush head was found to show the comparably highest load. This might be explained by its smooth, non-porous surface, allowing a certain stability of the droplets and a slower evaporation, resulting in less collapse of the envelope of the virus. This, however, remains speculative and cannot be finally confirmed by the current data. The toothpaste, brushing technique, the toothbrush type (design and number of bristles, powered toothbrush, etc.), and interactions with salivatory components may limit the generalizability of the findings.

The study only included traditional toothbrush. More in-depth research on dental hygiene routines that deviate from the lab standard applied in this study is needed, for example, with electric toothbrushes or toothbrushes made of different materials (e.g., bamboo or wood) or more frequent brushing cycles.

## Conclusions

The toothbrush appears to play an insignificant role in the (self-) transmission of coronavirus or influenza virus. Nevertheless, an appropriate use of oral health aids, i.e., using one toothbrush each person, avoid contact between the aids used by different individuals, etc., would be recommendable, regardless of the findings.
